# Synthesis, Bioapplications, and Toxicity Evaluation of Chitosan-Based Nanoparticles

**DOI:** 10.3390/ijms20225776

**Published:** 2019-11-16

**Authors:** Balsam R. Rizeq, Nadin N. Younes, Kashif Rasool, Gheyath K. Nasrallah

**Affiliations:** 1 Department of Biological and Environmental Sciences, College of Arts and Sciences, Qatar University, P.O. Box 2713, Doha, Qatar; br1512683@qu.edu.qa; 2 Biomedical Research Center, QU Health, Qatar University, P.O. Box 2713, Doha, Qatar; 3 Department of Biomedical Science, College of Health Sciences, QU Health, Qatar University, P.O. Box 2713, Doha, Qatar; ny1204022@qu.edu.qa; 4 Qatar Environment and Energy Research Institute (QEERI), Hamad Bin Khalifa University (HBKU), P.O. Box 5825, Doha, Qatar

**Keywords:** Chitosan, nanoparticles, biomedical, pharmaceuticals, toxicity

## Abstract

The development of advanced nanomaterials and technologies is essential in biomedical engineering to improve the quality of life. Chitosan-based nanomaterials are on the forefront and attract wide interest due to their versatile physicochemical characteristics such as biodegradability, biocompatibility, and non-toxicity, which play a promising role in biological applications. Chitosan and its derivatives are employed in several applications including pharmaceuticals and biomedical engineering. This article presents a comprehensive overview of recent advances in chitosan derivatives and nanoparticle synthesis, as well as emerging applications in medicine, tissue engineering, drug delivery, gene therapy, and cancer therapy. In addition to the applications, we critically review the main concerns and mitigation strategies related to chitosan bactericidal properties, toxicity/safety using tissue cultures and animal models, and also their potential environmental impact. At the end of this review, we also provide some of future directions and conclusions that are important for expanding the field of biomedical applications of the chitosan nanoparticles.

## 1. Introduction

Currently, the application of nanomaterials is gaining wider attention in pharmaceutical and biomedical research. Nanoparticles (NPs) of <100 nm in size present enhanced ability to get better patient compliance, enhanced biodistribution, and site-specific drug delivery [1]. Several advanced nanomaterials are employed in the biomedical and pharmaceutical industry. These advanced functional nanomaterials include magnetic nanoparticles [2], silica-based nanomaterials [3,4], metal and metal-oxide nanomaterials [5,6,7,8], and biological [9,10,11] and carbon nanostructures [12,13], to name a few. Engineered nanomaterial-based biomedical devices and biosensors can achieve a new level of sensitivity, selectivity, effectiveness, and biological stability for biological application. Additionally, nanomaterials are becoming an environmentally friendly and cost-effective option for efficient biomedical applications in gene delivery and transfection [14,15], as well as drug delivery carriers [16,17] and antibacterial agents [18,19,20,21], for wound healing [22], in nano systems against cancer [23], and as therapeutic delivery systems [24].

Uses of biopolymers such as starch, cellulose, silk fibroins, collagen, gelatin, albumin, and chitosan (Ch)-based nanomaterials endow the synthetic NPs with biocompatibility, biodegradability, and low toxicity. Biocompatible nanomaterials with high specific surface area are desirable in a plethora of biological and biomedical applications, such as drug delivery, therapeutics, and gene delivery. In recent years, several studies focused on the advances in this field, leading to substitute biocompatible nanomaterials considering the use of alternative resources, innovative properties, and limitations.

Ch is a natural linear polysaccharide cationic and hydrophilic polymer, obtained by alkaline hydrolysis of chitin; it is a non-toxic, biocompatible polymer consisting of randomly distributed β-(1, 4)-linked d-glucosamine (deacetylated) and *N*-acetyl-d-glucosamine (acetylated) units. Chitin is one of the most abundant natural amino polysaccharides obtained from the components of cell walls in fungi, and certain hard structures in invertebrates and fish. Ch has an abundance of hydroxyl (–OH) and amine (–NH_2_) functional groups, which can be employed to react with cross-linking agents for in situ chemical cross-linking. Ch is not only biocompatible and non-toxic; it is also biodegradable by certain enzymes into non-toxic oligosaccharides, making Ch appropriate for clinical use [25]. In the biomedical application arena, Ch-based nanomaterials revealed great success as antimicrobial agents, as well as for membrane separation, as carriers for drug delivery, as sensing materials for biomolecule monitoring, and in tissue engineering [26]. Additionally, Ch derivatives and Ch nanoparticles (ChNPs) depicted excellent performance in ophthalmology, dentistry, bio-imaging, bio-sensing, and diagnosis [27]. Historically, Ch, derivatives and ChNPs are among the most extensively studied class of natural biopolymer materials for biomedical applications.

ChNPs can be synthesized using either “bottom-up” or “top-down” approaches and/or combination of both procedures. Moreover, Ch-derivatives and ChNP composites are prepared to enhance the performance of the pristine Ch, such as reducing agglomeration and improving overall stability [28]. For instance, a supplement of DNA and RNA into mammalian cells by gene delivery can be used for treating diseases either to express new proteins or to prevent the expression of existing proteins [29]. Ch is used as a polycationic non-viral vector for gene delivery because of its biocompatibility and biodegradability; however, chemical modifications to its structure are required to efficiently and practically transfect under physiological conditions. To overcome this, encapsulated ChNPs, without the use of chemical modifications and organic solvents, are developed using a different synthesis technique [14]. Excellent gene delivery vehicles for in vivo applications were developed using these biocompatible Ch nanocomposites, proposing new insights into the field of non-viral gene therapy [14,29]. Structural modification or additive incorporation of Ch is also an efficient way to improve the stability of the polyplex in biological fluids, as well as enhance targeted cell delivery [30]. Moreover, Ch and its derivatives are among very few biomaterials that can be synthesized in large quantities while being economically viable. As discussed earlier, Ch is made of non-toxic monomeric units, and their environmental degradation leads to non-toxic by-products. Consequently, research on Ch and its derivatives found its niche in the main area of clinical research and biomedical applications.

This review presents an overview on the state of the art regarding Ch-based nanomaterials in the biomedical field. Special attention is dedicated to their preparation, properties, and application in cancer therapeutics, as well as their use as wound-healing dressings, as therapeutic delivery systems, and for drug delivery and transfection. The major concerns related to Ch stability in aqueous solution, as well as its antibacterial properties, antifungal properties, and toxicity, are addressed in order to understand the prospective of these materials in various biomedical applications. In addition to their bioapplications, we critically review the main concerns and mitigation strategies related to chitosan bactericidal properties, potential in vivo toxicity/safety, and their potential environmental impact.

## 2. Synthesis and Characterization

Ch consists of three functional groups, including an amino group and primary and secondary hydroxyl groups. Ch can be cross-linked with glutaraldehyde, glyoxal, and terephthaldehyde, producing hydrogels that can be used in different applications such as organ transplants, restoring organ function, gene delivery, and targeted therapy [31,32,33]. Ch has the advantage that a range of derivatives can be synthesized from Ch due to the existence of the amino group in conjunction with the primary alcohol function, which gives rise to *N*-modified Ch, *O*-modified Ch or *N*,*O*-modified Ch. The synthesis of Ch derivatives is usually performed to improve certain properties, such as quaternized and *N*-alkyl or *N*-benzyl Ch derivatives which can improve the antimicrobial activity of Ch, whereas phosphorylated Ch increases the antimicrobial activity and solubility [34]. Although several procedures are reported to describe a selective modification of Ch, there is a certain procedure which can be employed to perform nonselective modification of amino or hydroxyl groups. The modifications performed to synthesize Ch derivatives are primarily determined by the type of reactants used. Nonselective *N*,*O*-modified Ch derivatives are synthesized by the reaction of hydroxyl and/or amino groups with electrophiles like alkyl halides, acids, or iso(thio)cyanides. On the other hand, selectively *O*-modified Ch can be obtained via an easy and efficient method in which the protonation of amino groups is performed using an acidic solvent or additives like H_2_SO_4_ or MeSO_3_H. Protonation results in making alcohol function the most reactive functional group while protecting 90–99% of the amino group function. Nevertheless, strong acids result in partial depolymerization of Ch, affecting the biological activity. The amino groups can be further selectively modified via a quaternization reaction, reductive amination, or peptide coupling. The amino function can be protected before reacting with the alcohol group to create selectively *O*-modified Ch derivatives without affecting the molecular weight. Subsequently, *O*-modified Ch is obtained via a deprotection step of the amino function. *N*-modified Ch derivatives are selectively produced via a method in which alcohol function is protected [35,36]. Even though the number of reaction steps is increased because of the application of protecting groups, it results in a larger range of derivations without affecting the molecular weight. Furthermore, a selective coupling reaction with the amino and hydroxyl function can be performed with the selective *N*- or *O*-modified Ch to produce *N*,*O*-modified Ch derivatives.

**Figure 1:**
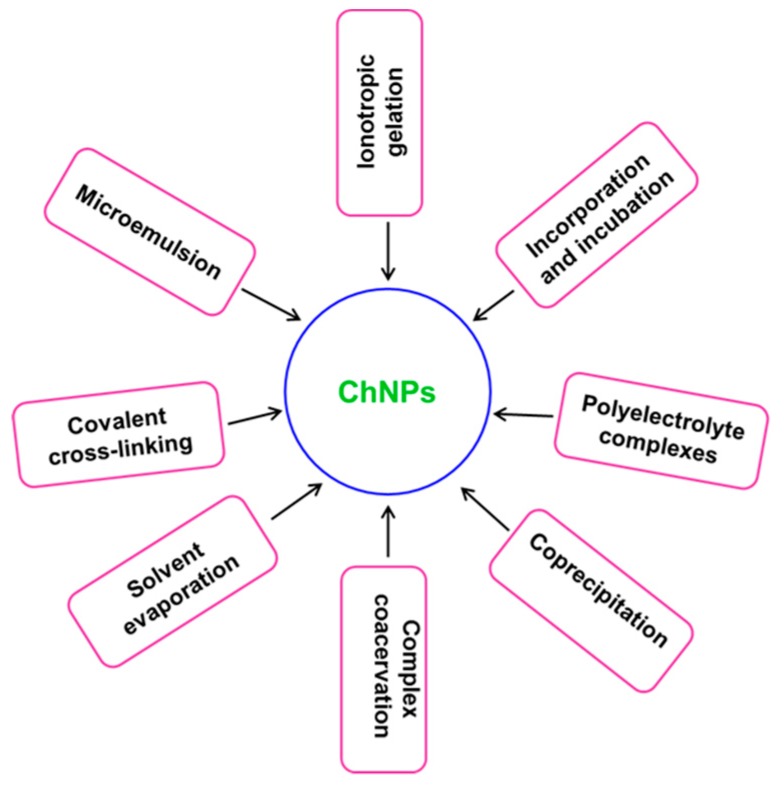
Schematic illustration of different methods of chitosan nanoparticle (ChNP) synthesis.

Recently, the development of ChNPs gained a lot of attention for a wide range of applications in the biomedical and pharmaceutical industry. NPs can be produced via “bottom-up” or “top-down” approaches or a combination of both techniques [33,37]. ChNPs are synthesized through numerous “bottom-up” approaches such as polymerization of Ch with methacrylic acid (PMAA) to make Ch-PMAA NPs, or through a reverse micelle medium or microemulsion methods [38]. Top-down approaches like milling, high-pressure homogenization, and ultra-sonication are also applied for the synthesis of these nanomaterials [33]. Figure 1 depicts the different methods applied for the synthesis of ChNPs.

**Figure 2:**
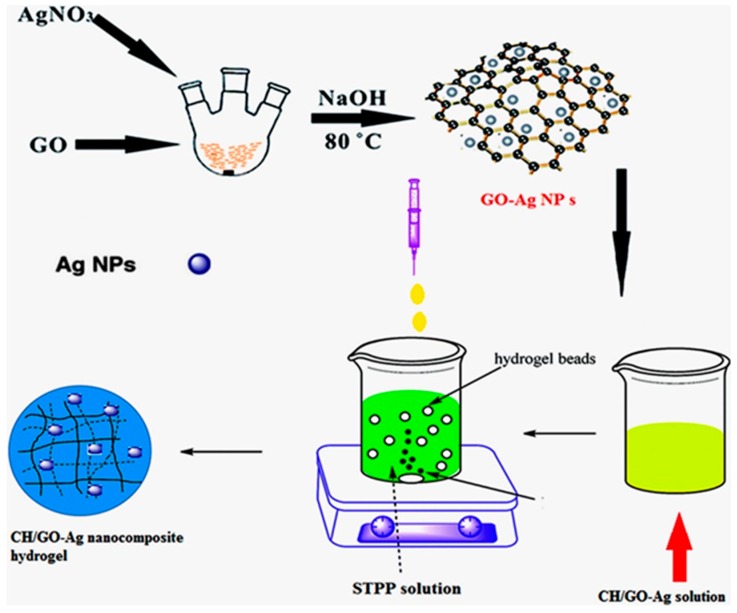
Schematic of facile preparation of bio-nanocomposite based on chitosan (Ch) and graphene oxide (GO) and silver (Ag) nanohybrids for controlled release of an anticancer drug [43].

At present, ChNPs are usually synthesized using a bottom-up ionic gelation method in which a solution of an anionic cross-linker, for example, sodium tripolyphosphate (TPP), and Ch is prepared where both reactants self-assemble into ChNPs because of the electrostatic interaction between the positively charged amine group of Ch and a negatively charged polyanion like TPP [14,37,39,40,41]. While several anionic cross-linkers like glutaraldehyde can be employed for ChNP synthesis, the use of TPP is more favorable because it is biocompatible and biodegradable [42]. NP size, hydrodynamic diameter, shape, and monodispersity were shown to be influenced by the Ch molecular weight (MW), initial concentrations of TPP and Ch, the Ch:TPP mass ratio, the degree of acetylation of Ch, and pH of the reaction media [37,40]. Ch-based bio-nanocomposites were synthesized using graphene oxide (GO) and silver nanohybrid particles for controlled drug delivery of, e.g., doxorubicin (Figure 2) [43]. It was also found that the presence of salts in the synthesis medium can highly influence the particle formation and average size [40]. In the microemulsion method, glutaraldehyde is added to a Ch acetic acid solution in the presence of a surfactant/hexane mixture under continuous stirring overnight [44].

Glutaraldehyde acts as a cross-linker in this method and reacts with free pendant amino groups of Ch to form stable imine bonds [45,46,47,48]. However, this method has the disadvantage of using glutaraldehyde, which is toxic to aquatic and other living organisms.

In a recent study, ChNPs were synthesized via spray freeze-drying as an alternative to the Ch–TPP cross-linking technique, and they exhibited size, structure, and viscoelastic behavior similar to that of Ch–TPP-synthesized NPs [38]. Usually, high-intensity ultra-sonication methods are applied to reduce overall particle size through a cavitation mechanism, in which particles are physically broken down to smaller sizes, or through polymer degradation initiated by a random scission model, which assumes that the scission of polymers occurs arbitrarily [49]. Ch is not soluble in water or other organic solvents; nevertheless, it is soluble in diluted aqueous acidic solutions with pH <6.5 and is usually amended by adding polyethylene glycol (PEG) or other cross-linking agents [50]. ChNPs can be synthesized via covalent cross-linking between Ch chains and agents like glutaraldehyde, PEG, and epichlorohydrin [51]. ChNPs for the delivery of protein molecules were produced via an incorporation and incubation method, in which a solution of Ch and protein was prepared, and then TPP was added to the protein–Ch solution leading to the formation of Ch–protein nanoparticles [52]. Ch-modified lipid-based nanoparticles via an emulsification/solvent evaporation method were synthesized for pharmaceutical applications [53,54]. The authors PEGylated the Ch chemoselectively, and precipitated it in a 15% Tris(hydroxyl methyl) amino methane aqueous solution. The precipitates were collected, washed, freeze-dried, and purified to yield Ch-grafted PEG free from any toxic residues [55]. Synthesized nanoparticles were applied as a carrier for pharmaceutical applications [53,55,56]. Highly monodisperse ChNPs and magnetic NPs were synthesized via coprecipitation of lactic-acid-grafted Ch to ammonium hydroxide and 6-mercaptopurine, and they were used for high drug loading and prolonged drug delivery [16,57]. Ch-coated Fe_3_O_4_ NPs were produced using an ex situ co-precipitation method in which Ch was cross-linked to Fe_3_O_4_ NPs through a combination of TPP/sulfate [58]. Complex coacervation was reported to produce ChNPs, improving their stability, biodegradability, photostability, oligomerization, and controlled release of bioactive compounds such as α-tocopherol (TOC), indole-3-carbinol, and 3,3′-diindolylmethane for drug delivery [59,60,61,62]. Several procedures, such as liquid–liquid phase separation, spray drying, chemical cross-linkers, ionic gelation, emulsion solvent diffusion, electrostatic interaction, and, most recently, ultrasound technology, were utilized to produce complex coacervates for different industrial applications [59,62,63,64,65,66].

The characterization of synthesized nanomaterials is vital to better understand the optimal formation of NPs and their influence on different applications. The physicochemical properties like size, shape, and surface morphology of ChNPs are well characterized and reported in the literature. The size of NPs is one of the most important factors that affect their applicability in the biomedical and pharmaceutical sector. For instance, smaller ChNPs possess better antibacterial activity and cell penetration than larger NPs and are even able to penetrate capillaries and tissue sinusoids for drug delivery [67]. The determination of ChNP size is usually challenging due to the polydispersity of the samples, and several complimentary procedures such as scanning electron microscopy (SEM), transmission electron microscopy (TEM), and atomic force microscopy (AFM) coupled with dynamic light scattering (DLS) are employed to determine the size of NPs [68,69,70,71]. DLS provides a hydrodynamic size and is more quantitative, while AFM and TEM offer both qualitative and quantitative information such as particle shape, surface morphology, and size of the NPs. Similarly, structural changes in the formation of ChNPs were reportedly observed employing TEM, AFM, SEM, Fourier-transform infrared spectroscopy (FTIR), and X-ray powder diffraction (XRD) [72,73,74,75,76].

## 3. Antimicrobial Aspects and Properties of Ch

Even with the enormous advancement in antimicrobial progression, the occurrence of antibiotic-resistant microbial strains dramatically enhanced, making antibiotic selections for infection control gradually inadequate and more costly. Antibiotic resistance could be assigned to several factors including evolution and advancement of resistant clones, and the insufficiency of current antimicrobial systems. Another reason is the suboptimal pharmacological characteristics of the ongoing antimicrobial structures, for which it is sometimes difficult to reach active concentrations inside bacterial strains or in some body sites [77]. Several natural antimicrobials were investigated and applied in the bio and pharmaceutical industry. Nevertheless, of these materials, Ch’s antimicrobial characteristics against a wide range of microorganisms, together with its comparative abundance and low cost, saw substantial growth in its applications in the biological and pharmaceutical industry [78]. Ch possesses significant antimicrobial activity against both Gram-negative and Gram-positive bacteria, as well as fungi [79,80].

### 3.1. Bactericidal Activity of Ch

The antibacterial activity of Ch is dependent on environmental factors such as pH of the media, type of pathogen, and on structural properties, namely, the degree of acetylation, MW, concentration, and source of Ch [81,82]. It was also stated that the quantity of Ch binding to the bacterial cell wall is reliant on the same factors [81]. Low environmental pH increases the positive charge in the Ch polymer, which increases its affinity for the bacterial cell wall [83]. Presumably, this is due to the increasing number of protonated amino groups on the polymer, where the positively charged –NH_3_^+^ groups promote binding to the negatively charged membrane components of the bacteria [82,84]. The antimicrobial activity is due to the fact that Ch is a cationic polyelectrolyte polymer. Low-molecular-weight Ch can go through microbial cellular areas, bind with DNA, and limit DNA interpretation and mRNA functions, while high-MW Ch can merge to the negatively charged components of the microbial cellular areas [85]. It forms an impermeable layer around the cell, changes cell permeability, and blocks transport into the cell. Microbes are capable of drastically hooking to the exterior of ChNPs in as short a period as 30 min; thus, ChNPs display antimicrobial activity [86]. The antimicrobial activity of Ch also depends on the type of microorganism [81]. ChNPs express more advanced interactions with Gram-negative bacteria than Gram-positive bacteria due to the former’s hydrophilicity and negative charge on their cell surface, which results in stronger antibacterial activity against them [70]. The polymer is able to exhibit activity against Gram-positive bacteria such as *Staphylococcus aureus, Bacillus cereus, Bacillus megaterium, Listeria monocytogenes, Lactobacillus plantarum, Lactobacillus bulgaricus*, and *Lactobacillus brevis* species. Ch is also effective against Gram-negative organisms, such as *Escherichia coli*, *Salmonella typhimurium*, *Pseudomonas fluorescens*, *Pseudomonas aeruginosa*, *Vibrio parahaemolyticus*, *Vibrio cholera*, and *Enterobacter aerogenes* [81,87].

A key challenge for the biomedical and clinical science fields is the risk of bacterial colonization of biomedical devices. To overcome this challenge, different nanomaterials are employed to produce antimicrobial coatings. Among them, Ch-based nanomaterials are already applied in several healthcare and industrial applications because of their non-cytotoxicity, biocompatibility, and excellent antimicrobial properties [88]. In order to enhance stability, antibacterial activity, and applicability, various Ch-based antibacterial nanocomposites were developed [89]. For instance, Chylińska and coauthors synthesized antibacterial Ch films modified with several hydantoin derivatives and their *N*-halamine analogues, and they reported that the introduction of biocides to the Ch enhanced the antibacterial activity of the coatings [90]. Several other studies reported the enhanced antibacterial activity of different Ch nanocomposites such as diisocyanate [89], quaternized [91], metal oxide [92], and carboxymethyl [93] modified Ch nanocomposites against both Gram-positive and Gram-negative bacteria. The synthesized nanocomposites can be used to prepare antibacterial coatings for a number of biomedical applications.

### 3.2. Antifungal Activity of Ch

The antifungal activity of Ch varies with the fungus due to the effects of MW and the degree of acetylation of Ch [94]. Ch exhibits antifungal activity against several phytopathogenic fungi including *Botrytis cinerea* in cucumber plants [78], *Penicillium* sp. in citrus fruit [95], *Alternaria solani* and *Fusarium oxysporum* in tomatoes [96], *Phytophthora infestans* [97], and others. Ch has antifungal properties against yeasts and molds, such as *Botrytis cinera*, *Fusarium oxysporum*, *Candida lambica*, *Rhizoctonia solani*, and *Phomopsis asparagi* [80]. This activity is believed to be fungistatic rather than fungicidal, inhibiting growth, spore germination, and tube elongation. The mechanism of action involves morphogenesis of the cell wall, which directly interferes with the growth. Additionally, Ch is believed to act faster on fungi than on bacteria [98]. The deacetylation (DA) of Ch influences the antimicrobial activity due to the number of free amino groups which contribute to the activity. Thus, it is believed that the activity increases as the DA decreases [99,100]. While investigating the activity of Ch from different sources, it was found that marine Ch possesses higher activity compared to fungal Ch obtained from *Rhizopus oryzae* [101]. In another study, however, crude fungal Ch from shiitake mushroom possessed higher antimicrobial activity than crustacean Ch [102]. There are different possible mechanisms regarding the mode of action. However, the exact mechanism is not fully understood, and many factors influence the activity, as stated above.

## 4. Biomedical Applications of ChNPs

Ch is a natural polysaccharide discovered 200 years ago (Braconnot) [103]. Ch has wide-ranging properties and characteristics that make it useful in a number of applications over a variety of fields. It is extremely versatile, non-toxic, biocompatible, and biodegradable, and, along with nanoparticles, it is utilized as a stabilizing agent because of its high permeability toward water, as well as its mechanical strength, film-forming ability, susceptibility to chemical modifications, and cost-effectiveness [104]. The biological properties of Ch and its derivatives enable them for versatile applications in the medical, pharmaceutical, nutrition, cosmetic, and food industries, as well as in water treatment, agriculture, and tissue engineering [82]. Table 1 reviews and outlines the many applications for Ch and examples of how Ch is used in those areas.

### 4.1. Chitosan Wound-Healing Activity

**Figure 3:**
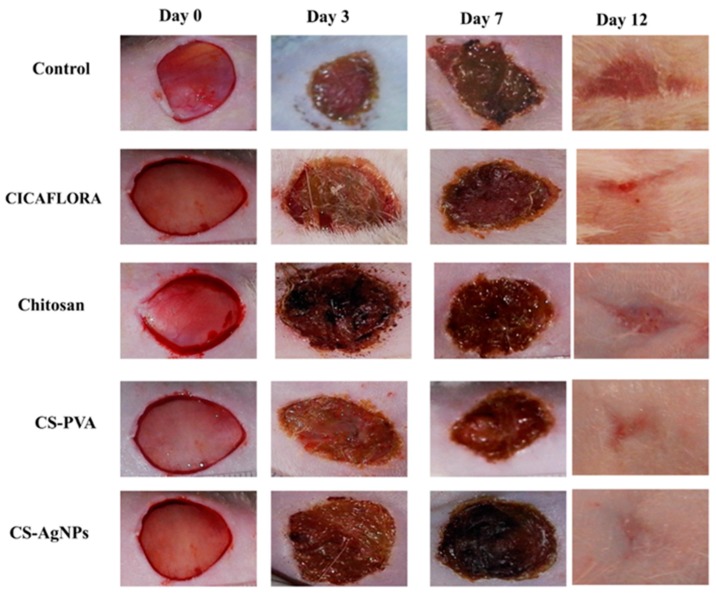
Representative photographs of the macroscopic appearance of wounds healing for groups treated with physiological serum (control), CICAFLORA^®^, chitosan (CS), CS–polyvinyl alcohol (PVA), and CS–silver nanoparticle (AgNP) gels [124].

Due to its antimicrobial, hemostatic, film-forming, anti-inflammatory, and analgesic activities, chitosan can be used as a wound-healing agent [109]. Ch can express antimicrobial activity in wound dressings in four forms, which are fiber, hydrogel, membrane, and sponge [84,113]. Most of the wound dressing materials exhibit their antimicrobial activity in a fabric form. Therefore, there is an interest in antimicrobial fibers, especially electrospinning techniques in which continuous polymer nanofibers are produced [114,115]. In a study by Chen et al., electrospun fibers composed of cross-linked collagen and Ch showed improved wound healing and tissue regeneration compared to gauze and collagen dressings [116]. Qasim et al. developed an electrospun Ch fiber with polyethylene oxide for periodontal disease and reported that the fibers could serve as surface layers mimicking local tissue structure and regenerating the wound site [117]. Sponges are flexible materials with good fluid absorption capacity and hydrophilicity, but they are mechanically weak in terms of holding their shape until new tissue forms. Thus, they can be used as burn dressing materials [113]. A Ch–gelatin sponge wound dressing was prepared and characterized. The sponge exhibited stronger antibacterial activity against *E. coli* K88 than penicillin and stronger activity against *Streptococcus* than cefradine. Additionally, the wound-healing time was found to be shorter compared to Vaseline sterile gauze [118,119]. Obara et al. prepared an insoluble and flexible hydrogel by applying ultraviolet irradiation to a photo-cross-linkable Ch solution containing fibroblast growth factor 2 (FGF-2) [120]. It was found that plain Ch hydrogel accelerated wound closure and wound contraction compared to no hydrogel treatment in both diabetic and normal mice. The hydrogel loaded with FGF-2 further accelerated the wound healing in the case of diabetic mice [120]. Chen et al. prepared a carboxy methyl Ch–alginate hydrogel integrated with gelatin microspheres and loaded with tetracycline hydrochloride. The in vitro drug release studies showed the sustained release of the tetracycline hydrochloride from the hydrogel [121]. Ch membranes are also promising materials. Azad et al. reported that a Ch mesh membrane shortened wound-healing time and promoted the recovery of the granular layer in a clinical and histological study [122]. A Ch–titanium dioxide composite membrane, which exhibited strong antibacterial activity against *S. aureus*, was prepared by Behera et al. Furthermore, the membranes decreased oxidative stress and apoptosis and showed rapid proliferation in the studied mouse fibroblast L929 cells [123]. Recently, Ch–polyvinyl alcohol (PVA)–silver nanoparticles were employed for wound-healing dressing and reported to stimulate the healing process as determined by the wound contraction ratio and histological examination [124]. Figure 3 depicts the wound-healing development for Ch and its composites when treating wound tissues as compared to a control. In another study conducted by Ma et al., Ch membranes were prepared loaded with drugs via a casting/evaporation method with the addition of glycerol, which provided a membrane with improved wettability, swelling rate, tensile strength, and water vapor permeability compared to a pure Ch membrane [125]. An in vivo study was conducted on 40 adult female albino rats by Ghannam et al. in which Ch nanosilver dressings were prepared and compared to the intradermal injection of mesenchymal stem cells. They reported that the non-invasive Ch nanosilver dressings exhibited faster and better wound-healing capabilities compared to mesenchymal stem-cell injections [126]. Ch is also used to prevent tissue adhesion in internal surgery. Ideally, these internal dressings should bioerode and be reabsorbed into the body when their purpose is completed. Grafted Ch filled this niche. Additionally, taking advantage of the non-toxicity, Ch is used in sutures for patients [27,127].

### 4.2. Chitosan-Based Nanosystems Against Cancer

Concerning cancer delivery, there were many published applications for Ch-based nanosystems in different cancers such as breast [128], colon [129], lung [130], brain [131], and others. Venkatesan et al., in 2011, presented promising outcomes from mouse–human xenograft models for the use of a hydroxyapatite–Ch nanosystem as a transporter and delivery agent for celecoxib and other drugs, aiming to treat colon cancer [132]. In addition, in 2012, Xu et al. published potential results for a ChNP modified with tripolyphosphate (TPP) to deliver interleukin-12 (IL-12) [133].

ChNPs possess interesting biomedical applications. They can be used as carriers in the controlled drug delivery of doxorubicin (DOX), an anticancer drug used for the treatment of several tumors. DOX is generally used in the treatment of several cancers such as acute leukemia, lymphomas, soft-tissue and osteogenic sarcomas, pediatric malignancies, and adult solid tumors such as breast and lung carcinomas. It is also used with other drugs such as methotrexate, cisplatin, ifosfamide, vincristine, and etoposide [134]. However, only a small amount of DOX reaches the tumor target site because about 40% is excreted via liver metabolism. Furthermore, DOX induces cardiac toxicity; for some patients, this started within one year of DOX therapy, while, for others, it occurred 15 years after the end of the treatment [135,136,137]. A solution to protect patients from DOX side effects is by using a drug delivery system compound with ChNPs. Due to the Ch properties of non-toxicity, biocompatibility, and biodegradability, it is possible to encapsulate and deliver DOX with reduced side effects. Furthermore, biodegradable doxorubicin conjugated with a Ch oligosaccharide showed high efficiency in the suppression of tumor growth due to higher cellular uptake [138,139].

Moreover, Ch drug delivery also includes 5-fluorouracil (5-FU) and leucovorin (LV), which are drugs used in the treatment of colon cancer [135], as well as avidin and biotin, which are drugs used for hepatic carcinoma treatment [140]. ChNPs were also studied in gene delivery systems [141], small interfering RNA (siRNA) delivery [111], release of vitamin C [142], delivery of plasmid DNA (pDNA) against hepatitis B through nasal mucosa [143], and protein delivery systems [112], such as insulin [144] or bovine serum albumin (BSA) delivery [145]; they were also used to enhance the absorption of polyphenolic antioxidants, for instance, catechins in the intestine [146]. Table 2 summarizes other uses for Ch and/or its derivatives as anticancer agents.

Given the high availability, low production cost, and valuable properties of Ch regarding low metabolic and immunogenic toxicity, it is repeatedly described in the literature as a suitable delivery system in several different fields and applications. It is considered very promising regarding cancer because of its muco-adhesivity, tending to selectively accumulate in mucus, preferentially at anionic cancer cell surfaces due to its cationic nature [129,153,154]. Other published characteristics are related to its use in tumor growth suppression, as an immune system adjuvant, and for its anti-inflammatory activity, which confer an anti-tumoral contribution to this compound [155,156]. Two major draw backs in Ch bioapplications are its low solubility at physiologic pH (~7.4) and its fast dissolution in the stomach [157]. At acidic pH (below the chitosan pK_a_ which is 6.3), the weakening of inter-chain interactions due to amino protonation leads to Ch dissolution. This behavior can be controlled by using derivatives or combined systems [158]. On the other hand, the pH sensibility of Ch can be an advantage in terms of loading and strategic delivery of drugs, for instance, in preventing the drug release at physiologic pH and promoting its preferential release in a tumor acidic environment or in liposomes or endosomes, as in the case of internalization [159,160].

### 4.3. Chitosan in Drug Delivery

In the area of drug delivery and therapeutics, nanoparticles are fabricated into drug delivery platforms for the treatment of a broad range of diseases, as well as scaffolds for tissue engineering. Chitosan is one of the most popular natural polymers with wide application in the discipline of drug delivery due to its cationic functionality and aqueous medium solubility [161]. The elimination of Ch after the delivery is easy through renal clearance; however, this applies only to Ch with a suitable molecular weight. Enzyme degradation is required for Ch with a very large molecular weight.

Nanomaterials or nanoparticles offer new opportunities in material science and biomedicine. The small size of nanoparticles allows them to enter cells and organelles, offering innovative approaches such as targeted drug delivery [162]. NP surfaces can be conjugated with ligands or antibodies that enable recognition and binding to specific receptors on the target cells [163]. Most interactions of nanoparticles with cell membrane proteins are non-specific in nature. While this allows nanoparticles to attach to cell surfaces [164], targeting to a specific organ or region of the body should require the modification of the surface property of the nanoparticles. The absorption and bioavailability of drugs encapsulated into ChNPs can be improved, allowing them to be used to deliver gene and protein drugs, as well as effectively protecting them from enzyme degradation in vivo [165]. It was shown that blood capillaries were capable enough to administrate Ch intravenously. However, the biodistribution of Ch can vary depending on the size, surface charge, and hydrophobicity of Ch and its derivatives [166].

Other applications of Ch involve the use of Ch surface coatings to improve the biocompatibility of other nanoparticles [167]; for instance, Ch was chemically modified with the hydrophobic *n*-hexanoic anhydride to form an amphiphilic Ch derivative that showed better blood compatibility [168]. Furthermore, the synthesis of surface Ch was modified in order to achieve desired therapeutic outcomes, e.g., targeted drug delivery [169]. For example, silver-loaded silicon dioxide nanoparticles coated with Ch (20 ± 5 nm) exhibited greater stability [170], as the Ch coating prevented the oxidation of the silver ions to black silver oxides, and this improved the antibacterial activity of the nanoparticles.

Other research showed that ChNPs loaded with insulin were also developed to improve the systemic delivery of insulin through the nasal passage [144]. The NPs were shown to reduce blood glucose levels by 52.9% in rats and 72.6% in sheep, but these response rates were no better than those observed for insulin dissolved in a Ch solution (40.1% in rat, 53.0% in sheep) [171]. Nonetheless, this study did demonstrate the potential of ChNPs to translocate through the nasal epithelia into systemic circulation.

The objectives of the most prominent applications of Ch nanoparticles in drug delivery are often to reduce drug side effects, control the rate of drug delivery, and ensuring that only the targeted area is treated [172]. For example, metronidazole (MZ) is an antibiotic with common side effects of nausea, vomiting, epigastric pain, and mouth dryness, most likely caused by high concentrations of residual MZ in the saliva [172]. To protect MZ from dissolution in saliva, the drug was loaded into ChNPs of 200–300 nm in size, showing a controlled release of the drug over 12 h in phosphate-buffered saline (pH 7.4) [173,174]. Drug dissolution within 1 h was reduced from 53% to 30–40 % after entrapment in the NPs.

ChNPs are developing into an important component in the field of polymeric therapeutic conveyance for the advancement of pharmacological and medicinal release to the targeted site, due to their good biodistribution, as well as their elevated specificity and sensitivity.

### 4.4. Chitosan As A Therapeutic Delivery System

Conveyance of the medicinal drug to a specific place in the body is a major complication in the remedy of various diseases [175]. Employing a drug delivery entity for already designed medicinal therapeutics provides an improvement in performance in terms of efficiency, safety of the patient, and a reduction in the number of side effects. Drug delivery entities built using polymers can boost the pharmacokinetics of the drug, advance the therapeutic index, lower the side effects, and accordingly escalate the resourcefulness of the entire system [176]. As a therapeutic delivery entity, ChNPs attract attention due to their relevance in storing protein therapeutics, as well as genetic and adverse tumor chemical therapeutics, by means of different pathways of intake such as oral, nasal, and intravenous [177]. The affected site-specific delivery of this Ch therapeutic conveyance entity is exceptionally greater due to the positive charge of NPs, which gives them the advantage of high affinity for negatively charged cell membranes [178]. The hydrophobic nature of Ch influences the efficient encapsulation of hydrophilic therapeutics into the ChNPs [161]. Another advantage of ChNPs is that Ch can increase drug permeability across absorptive epithelia by disrupting the intercellular tight junctions through the transport of tight junction proteins from the plasma membrane to the cytoskeleton [179,180]. As an anti-inflammatory, Ch acquires its anti-inflammatory action based on its acid hydrolysis to glucosamine hydrochloride and its derivatives. These monosaccharides found in connective tissues and cartilage are the structural units of proteoglycans. By absorbing these monosaccharides, damaged or inflamed tissues can be restored and regenerated [181,182]. This mechanism can be considered as an active treatment for bone hyperplasia and rheumatoid arthritis. Moreover, in comparison to some typical analgesic and anti-inflammatory drugs and anti-arthritic steroidal drugs, these monosaccharides are considered safe, as they have no harmful side effects in the long term. Some experiments revealed that glucosamine treatment for two weeks can improve movement and eliminate arthritic pain in patients suffering from severe arthritis [181]. Indeed, the mechanism of the analgesic effect was investigated using an acetic-acid-induced writhing test on mice using a Ch suspension mixed with a 0.5% acetic acid solution. The results suggested that the analgesic effects of Ch treatment were due to the absorption of proton ions from the inflammatory site, causing a pH increase [183].

### 4.5. Chitosan in Gene Delivery and Transfection

ChNPs are able to deliver biologically active materials into cells without compromising the integrity of the cargo or the cell, as the NPs are internalized into the cells via endocytosis [184]. Thus, Ch–DNA complexes of 50–100 nm in size were efficiently transfected into HeLa cells within an hour of exposure without associated cellular toxicity at concentrations of 100 μL/mL. The control polyethylenimine–DNA complexes at the same concentrations were seen to induce cytotoxicity [15]. This is an important advantage as the advent of biopharmaceuticals requires innocuous delivery systems that can protect sensitive biologics, such as proteins and genes, against enzymatic and chemical degradation [174,185]. In gene delivery, ChNPs can inter-react with negatively charged DNA and transform into a polyelectrolyte complex. Nuclease degradation was found to be ineffective when DNA was included in these complexes, leading to better transfection efficiency [186].

The transfection efficiency of Ch-based nano-vehicles is cell-type-dependent; there is no toxicity with respect to other more toxic particles like lipofectamine, a cationic lipid; unfortunately, the transfection efficiency of Ch nanoparticles is lower than that of lipofectamine [187]. For an effective transfection, not only is the internalization important, but so is the subsequent endo-lysosomal escape [188]. Ch was chosen for this task since it can exploit its buffer capacity in a restricted interval of pH values (5–7), and promote the rupture of the endosomes after 72 h with consequent escape into the cytosol [189]. Yu et al. synthesized a copolymer of poly (l-lysine) with Ch and studied its efficiency in relation to plasmid DNA adherence capability, as well as its gene transfection effect in HEK 293T cells, compared to a pristine Ch polymer [190]. In conclusion, we can state that Ch has wide-ranging applications as a drug and gene carrier.

## 5. Evaluation of Toxicity

Chitosan nanoparticles emerged as a pivotal instrument in many fields, including chemistry, water treatment, aquatic herbicides, bioengineering, disease detection, and drug delivery. In this context, the wide range of ChNP applications necessitates accurately investigating the potential ChNP toxicity for both aquatic life and higher vertebrate animals. Below, we summarize and critique the main findings of studies that used the zebrafish model to assess the toxicity/safety of chitosan nanoparticles. We describe the toxicity of different Ch nanocomposites using zebrafish embryos at multiple levels, including mortality, teratogenicity, organ-specific toxicity, and genotoxicity.

**Figure 4:**
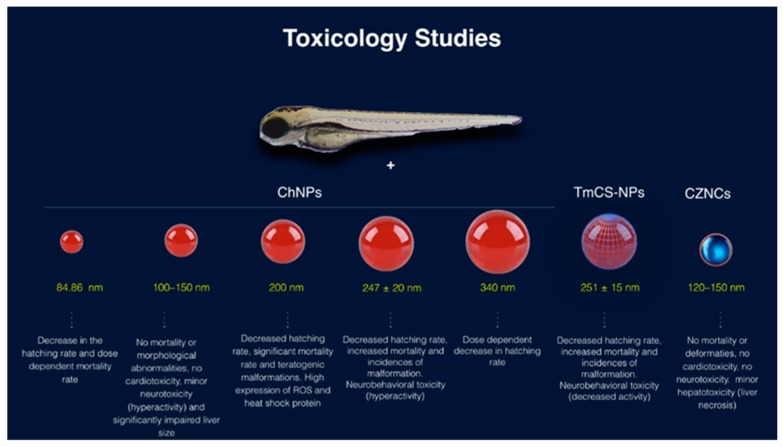
Summary figure representing the toxicity of different sizes of ChNPs, Tween modified Ch and Ch zinc oxide nanoparticles on zebrafish embryo. 4 studies investigated the toxicity of different sizes of ChNPs (100–340 nm). The smallest ChNPs tested for its toxicity sized 84.86 nm, in which it showed dose-dependent increase in mortality rate (LC50 280 mg/L), decrease in the hatching rate. 200 mg/L of ChNPs size ranging from 100 to 150 nm showed minor neurotoxicity (hyperactivity) and liver necrosis. 30 mg/L of ChNPs size 200 nm showed a significant decrease in the hatching rate. 12 mg/L of ChNPs size 247 nm showed significantly decreased hatching rate, increased mortality and neurobehavioral toxicity (hyperactivity). 20 mg/L of ChNPs size 340 nm showed significantly decreased hatching rate. The Tween modified ChNPs (TmCS-NPs) size 251 nm showed significantly decreased hatching rate as well. However, it showed a decrease in neural activity (Neurobehavioral toxicity) at very low concentration as 12 mg/L. Finally, Ch/zinc-oxide nanoparticles (CZNC) size 120 to 150 nm showed only minor hepatotoxicity (liver necrosis) at only high concentrating (250 mg/L).

ChNPs show promising results for in vivo use as drug delivery vehicles and diagnostic materials. However, it is essential to understand how NPs interact with cells and organs to ensure their safety with respect to clinical or environmental exposure. Zebrafish embryos were used in our and other laboratories as an in vivo model to evaluate nanoparticle biocompatibility. The zebrafish model can be used to assess nanoparticle toxicity at multiple toxicity levels, including the mortality rate, teratogenic effect, neurotoxicity, hepatotoxicity, and genotoxicity. This model was utilized in different Ch nanotoxicology studies [191,192,193,194,195]. The toxicities of different sizes of ChNPs, Tween-modified Ch, and Ch/zinc-oxide NPs were investigated using zebrafish embryos (summarized in Figure 4). Five articles studying the effect of ChNPs on zebrafish embryos were published and are summarized here in Table 3 [191,193,194,195]. Yuan and his collogues studied the toxicity of ChNPs and their Tween-80-modified counterparts using zebrafish embryos [195]. These nanoparticles are two of the most commonly used brain-targeted drug vehicles. Yuan et al. showed that Tween-80-modified ChNPs (TmCS-NPs) induced developmental toxicity in the embryos, including a decrease in hatching rate, as well as an increase in the mortality and in the incidence of deformities, in a dose-dependent manner. In addition, both nanoparticles induced neurobehavioral toxicity, including decreased spontaneous movement in TmCS-NP-treated embryos and a hyperactive effect in ChNP-treated embryos. Moreover, both NPs inhibited axonal development of the motor neurons and remarkably affected the muscle structure of the embryos. However, Yuan et al. could not experimentally rule out that this toxicity was potentially related to the remaining traces of Tween-80 after Ch modification. From our experience, we know that traces of chemical contamination such as acetic acid can cause drastic mortality to zebrafish embryos.

The second study was conducted by Hu and his colleagues to test the potential ChNP toxicity in relation to particle size, whereby they tested the toxicity of 200-nm and 340-nm ChNPs using zebrafish embryos. Hu et al. reported that 200-nm ChNPs were more toxic compared to the larger nanoparticles (340 nm). The smaller nanoparticles were able to cause 100% mortality to the embryos and severe teratogenic phenotypes at very low concentration (40 mg/L). On the other hand, 340-nm ChNPs, albeit to a lesser extent compared to the smaller nanoparticle, were able to promote significant mortality and teratogenic phenotypes [194]. Hu and his colleagues also reported that the 200-nm ChNPs caused teratogenic deformities including bent spine, pericardial edema, and an opaque yolk in zebrafish embryos. Moreover, they reported that ChNP-treated embryos showed a significant increase in cell death rate and increased presence of reactive oxygen species.

The last two studies were in disagreement with Hu and his colleagues’ results. Wang et al. showed that 200 mg/L ChNPs failed to induce significant mortality (<10%) to the treated embryos, even when employing a smaller nanoparticle size of Ch (84.86 nm). In addition, Abou-Saleh et al. employed smaller ChNP sizes (100–150 nm) than those used by Hu et al., and no toxic effects or teratogenic phenotypes were recorded at concentrations as high as 200 mg/L. Abou-Saleh et al. were the first to comprehensively study the organ-specific toxicity of ChNPs in zebrafish embryos. They reported that embryo treatment with ChNPs was unable to induce embryo deformities or mortality at the used concentrations. In addition, ChNP-treated embryos displayed normal heart physiology, including normal heartbeat frequency, normal corrected QT interval (QTc), and normal ejection fraction. Furthermore, ChNP-treated embryos at high concentration showed an abnormal hyperactivity compared to the negative control and a significant impairment of the liver size. Thus, Abou-Saleh et al. suggested that ChNPs at high concentrations might be potentially toxic to zebrafish embryos [193].

One of the essential applications of ChNPs is combating marine biofouling. Recently, a new Ch/zinc-oxide nanoparticles (CZNC) composite was used as a promising “green” biocide. Due to the eco-friendly nature of Ch, it provides a novel pathway to develop less toxic biocides for combating marine fouling without affecting the aquatic fauna. Younes et al. showed that exposure of zebrafish to 25–200 mg/L CZNCs did not cause any significant signs of toxicity or deformities to the treated embryos. They confirmed their results by performing cardiotoxicity assays in which CZNC-treated embryos showed a normal heartbeat frequency, rhythmic activity, and contractile functions. Furthermore, the neurotoxicity assay revealed that CZNC-treated embryos did not elicit any significant impact on the neurological behavior of the embryos. Finally, the hepatotoxicity assay showed that CZNCs might have a mild toxic effect on the liver [192].

## 6. Future Outlook and Conclusions

Ch-based nanomaterials are among the most promising polysaccharide biomaterials being synthesized for different applications, because of their distinctive characteristics, biodegradability, non-toxicity, and antimicrobial properties. To expand their applicability, a broad understanding of their activity is essential. ChNPs are extensively studied for biological, biomedical, and pharmaceutical applications, including drug delivery and gene delivery, as well as a therapeutic delivery system and nanosystem for cancer, for wound healing, and as bactericidal agents. Current research is focused on improving the stability, biocompatibility, and synthesis of novel ChNPs to enhance their effectiveness in biomedical applications. Ch modifications can be carried out, and derivatives can be developed by tuning and controlling the surface chemistry, including chemical modifications via the hydroxyl and amino groups using chemical reactions like cross-linking, carboxymethylation, etherification, and graft copolymerization, to name a few. Similarly, several ChNPs and nanocomposites with polymeric matrices, such as polyglycolic acid, polylactic acid, metal and metal oxides, and carbon nanostructures, were developed to overcome a few limitations of pristine ChNPs, including aggregation, solubility, and antimicrobial activity. Considering the targeted applications, these nanomaterials can be applied in a solubilized form, such as suspensions, coatings, hydrogels, and films, and they show high potential for antitumor applications, protein and peptide drug delivery, cardiovascular applications, bone reconstruction, blood purification, cancer treatment, and tissue regeneration applications. Ch is biodegradable due to its depolymerization by the bacterial enzyme chitinase and lysozyme, resulting in monomers of glucosamine and *N*-acetylglucosamine. The application of ChNPs in the biological and biomedical fields is due to the biocompatibility and biodegradability of Ch. Due to its cationic characteristic and primary amino groups, Ch is among one of the most important polysaccharides for several drug delivery purposes, including controlled drug release, in situ gelation, and transfection. Additionally, as Ch is made from a naturally abundant biopolymer, it may be a good choice for cost-effective biomedical applications. Because of its biocompatibility and antimicrobial activity, Ch is used as a bactericidal and anti-fungal agent, and as a coating in a wide variety of biomedical and industrial applications.

While the use of nanomaterials offers great advantages in the biomedical field, current research on the safety of various NPs is not enough for their application in the biomedical field. Commonly, Ch was found to be relatively safe due to its biodegradable and biocompatible properties. However, several studies showed the cytotoxicity of ChNPs in vitro and in vivo. Thus, the present knowledge on Ch-based nanomaterials is not developed enough, and extensive research on the fabrication of ChNPs and their biological properties is urgently needed. In particular, more research is required to comprehensively investigate the toxicity of ChNPs for human beings and other living organisms. Moreover, green and environmentally benign synthesis methods for Ch derivatives should be developed to protect the environment. Nevertheless, despite a few shortcomings, ChNPs are considered promising materials for biomedical applications.

## Figures and Tables

**Table 1:** Field of application potentials of chitosan (Ch) and its derivatives [105].

Applications	Functions	References
Antimicrobial agent	Bactericidal and fungistatic	[98,106]
Food industry	Preservative, food stabilizer, gelling agent, food additive, controlled enzymatic browning in fruits, controlled release of antioxidants, controlled moisture, temperature control, color stabilization, etc.	[61,82]
Biotechnology	Protein separation, chromatographic media, enzyme immobilization, catalyst, imaging, dialysis, filtration, etc.	[78,107]
Agriculture	Fertilizer, seed coating, etc.	[82,108]
Medical applications	Clotting agent, wound healing and tissue engineering, skin burn, surgical sutures, blood cholesterol control, antitumor agent, membranes and scaffolds, etc.	[14,23,26,109]
Cosmetics	Skin and hair products	[110]
Delivery	Controlled drug delivery, gene delivery, oral peptide and protein delivery, small interfering RNA (siRNA) delivery, etc.	[1,31,32,33,111,112]

**Table 2:** A summary of Ch and its derivatives with their most important anticancer activities [78].

Chitosan and Its Derivatives	In Vitro Cell Lines and In Vivo Models	Function	References
Carboxymethyl chitosan	BEL-7402 cell line Hepatoma cell line H22 in mice model	- Inhibited lung metastasis in mouse model - Reduced the expression of MMP-9	[147]
Carboxymethyl chitosan	Apoptosis models in Schwann cells using hydrogen peroxide induction	- Carboxymethyl chitosan, increased Bcl-2 activity and decreased Bax, caspase-3, and caspase-9 activities - Improvement of the cell viability	[148]
Chitosan	RPMI7951, SKMEL28, and A375	Chitosan was coated in culture wells of RPMI7951, SKMEL28, and A375. - In RPMI7951, induction of CD95 receptor expression which induced FasL apoptosis. - In SKMEL28 cells, decreased proliferation - In A375 cells, decreased adhesion	[149]
Chitosan	Transplantation of meth-A solid tumor in BALBc mice	Interleukin 1 and 2 induction and proliferation of cytolytic T lymphocytes, enhancing the anticancer activity	[150]
Chitosan	LCC and HepG2 cell line xenografts in mouse model	- S-phase arrest and inhibition of DNA synthesis - Downregulation of CDK-2 and cyclin A, upregulation of p21, and inhibition of MMP-9 expression in order to decrease metastasis and inhibit tumor growth	[151]
Chitosan	HepG2, A549, and PC3 cell line	Suppression of HepG2, A549, and PC3 cancer cell growth via 50% cell death	[152]

MMP-9: Matrix metallopeptidase 9; Bcl-2: B-cell lymphoma 2; Bax: Bcl-2-associated X protein; CD95: Cell adhesion 95 also known as Fas; FasL: Fas ligand; S-phase: DNA synthesis phase in cell cycle; CDK-2: Cyclin-dependent kinase 2.

**Table 3:** Summary of the five studies of chitosan nanoparticle toxicity using a zebrafish model.

Nanoparticle	LC_50_ (mg/L)	Particle Size	Teratogenicity	Assays	Reference
ChNPs	23.26 mg/L	247 ± 20 nm	Uninflated swim bladder and bent spine	Mortality rate, hatching rate, malformations, neurobehavioral activity assessments and apoptosis assay	[195]
Tween modified ChNPs (TmCS-NPs)	25.06 mg/L	251 ± 15 nm	Uninflated swim bladder and bent spine		[195]
ChNPs	Not recorded	200 nm	Dose-dependent decrease in hatching rate; malformations including a bent spine, pericardial edema, and an opaque yolk in zebrafish embryos; increase in heat-shock protein	Acridine orange staining and Western blot	[194]
ChNPs	Not-Recorded	340 nm	Dose-dependent decrease in hatching rate	Acridine orange staining and Western blot	[194]
ChNPs	>200 mg/L	100–150 nm	no mortality, but morphological abnormalities; neurotoxic effects and significant impairment of liver size	Organ-specific toxicity (cardiac, hepatic, and neuromuscular)	[193]
ChNPs	280 mg/L	84.86 nm	Decrease in the hatching rate and dose-dependent mortality rate	Mortality rate and hatching rate	[191]
Ch/zinc-oxide nanoparticles (CZNC)	>250 mg/L	120–150 nm	No cardiotoxic or neurotoxic effects and minor hepatotoxic effect	Organ-specific toxicity (cardiac, hepatic, and neuromuscular)	[192]
